# 

**Published:** 1871-09

**Authors:** 


PUBLISHED MONTHLY, AT $3 PER ANNUM, IN ADVANCE.

### ATLANTA

### Medical and Surgical Journal.

EDITED BY

W. F. WESTMORELAND, M. D.,

*Professor of the Principles and Practice of Surgery in Atlanta Medical College.*

AND

J. G. WESTMORELAND, M. D.,
*Professor of Materia Medica and Therapeutics in Atlanta Medical College.*

*Pax et Scientia, sed veritas sine timore.*

VOLUME IX[illegible]—SEPTEMBER, 1871.—NUMBER 6.

ATLANTA, GEORGIA:
PLANTATION PUBLISHING COMPANY.
1871.

EACH NUMBER CONTAINS SIXTY-FOUR PAGES.

